# Feasibility and preliminary efficacy of guided internet-delivered cognitive behavioral therapy for insomnia after the loss of a child to cancer: Randomized controlled trial

**DOI:** 10.1016/j.invent.2021.100409

**Published:** 2021-06-05

**Authors:** Josefin Sveen, Susanna Jernelöv, Lilian Pohlkamp, Ulrika Kreicbergs, Viktor Kaldo

**Affiliations:** a Palliative Research Centre, Department of Health Care Sciences, Ersta Sköndal Bräcke University College, Stockholm, Sweden; b National Center for Disaster Psychiatry, Department of Neuroscience, Uppsala University, Uppsala, Sweden; c Division of Psychology, Department of Clinical Neuroscience, Karolinska Institutet, Stockholm, Sweden; d Centre for Psychiatry Research, Department of Clinical Neuroscience, Karolinska Institutet, Stockholm Health Care Services, Region Stockholm, Sweden; e Department of Women's and Children's Health, Paediatric Oncology and Haematology, Karolinska Institutet, Stockholm, Sweden; f Department of Psychology, Faculty of Health and Life Sciences, Linnaeus University, Växjö, Sweden

**Keywords:** Bereavement, Child loss, Treatment, Internet intervention

## Abstract

Bereaved individuals often experience sleep problems. The aim of this study was to evaluate feasibility and preliminary effects of internet-delivered cognitive behavioral therapy for insomnia (iCBT-i) in bereaved parents. Parents were randomized to iCBT-i (n = 10) or an active control group (n = 11). Primary outcome (insomnia) and secondary outcomes (prolonged grief, depression, posttraumatic stress, and grief rumination) were assessed pre- and post-treatment, with 9- and 18-month follow-ups. Feasibility was assessed post-treatment and one month later. Most parents reported positive effects of the treatment. The intervention group improved significantly from pre- to post-treatment and had a significantly larger reduction of insomnia when analyzed over all four time-points (Wald χ^2^ = 30.0, *p* < 0.001), although the effect at post-treatment was very small (*d* = 0.1) for insomnia. Thus, iCBT-i was feasible and was related to reduced insomnia and psychological distress in bereaved parents, both short- and long-term, but the results regarding the treatment effect are preliminary due to the small sample size.

## Highlights

• The online CBT for insomnia was feasible in bereaved parents.
• No adverse effects were reported by the bereaved parents.
• The online CBT for insomnia effectively alleviated insomnia in bereaved parents.
• The insomnia treatment had a positive effect on reducing psychological distress.

## 1 Introduction

About 20% of children with cancer in Western countries do not survive their disease, making cancer one of the leading causes of death among children over one year of age (Rosenberg et al., 2012; Swedish Board of Health and Welfare, 2016). Bereaved parents are at increased risk of developing mental and physical health problems (Stroebe et al., 2007) and bereavement is also associated with an increased risk of mortality, especially in mothers (Li et al., 2003). A systematic review (Rosenberg et al., 2012) reported that parents of children who die from cancer have higher rates of anxiety, depression, and prolonged grief, and poorer quality of life, compared with other parents.

Impaired sleep is a common problem in bereaved individuals (Buckley et al., 2012; Hardison et al., 2005; Lancel et al., 2020). However, there are few studies examining sleep impairments after the loss of a child (Lancel et al., 2020). One previous study (Lannen et al., 2008) has reported that a majority (73.5%) of parents who had lost a child to cancer had sleep difficulties 4–9 years later.

There is strong evidence that sleep difficulties is associated with mental health problems following loss, such as prolonged grief and depression (Boelen and Lancee, 2013; Lancel et al., 2020; Monk et al., 2008). For example, several studies have shown that more severe sleep difficulties are associated with higher levels of prolonged grief (Boelen and Lancee, 2013; Germain et al., 2005; Hardison et al., 2005). A study targeting treatment of prolonged grief found a modest improvement in sleep quality, but the sleep difficulties remained after treatment even though prolonged grief symptoms had decreased (Germain et al., 2006). This suggests that insomnia (a sleep disorder characterized by subjective difficulties with sleep onset and/or maintenance) is a partly independent factor, requiring specific treatment in individuals with prolonged grief, much like what has been seen for insomnia in relation to depression (Blom et al., 2015a). In addition, persistent sleep deprivation can increase the risks for a variety of negative health outcomes (McEwen, 2006). Thus, treating insomnia may improve not only sleep, but also health in general.

Despite insomnia being common in bereaved individuals, only one pilot study (Carter et al., 2009) has evaluated feasibility and preliminary efficacy of an insomnia treatment – a brief two-session home based cognitive behavioral therapy (CBT) – in cancer-bereaved caregivers. Eleven participants were included in two 2-hour sessions of a 5-week study protocol taking place at the participants' home, with focus on identifying and changing sleep habits. Preliminary results indicated improved sleep and reduced levels of distress, but the absence of a control group limits the interpretation of the results and more rigorous research is needed in bereaved individuals examining the effect of cognitive behavioral therapy for insomnia (CBT-i). CBT-i is the recommended first-line treatment for adults (Riemann et al., 2017; Wilson et al., 2019). CBT-i has been shown to be effective in decreasing insomnia severity and improving sleep efficiency also when delivered via the internet (Blom et al., 2015a; Blom et al., 2015b; Kaldo et al., 2015; Seyffert et al., 2016), with effect sizes similar to those of face-to-face therapy (Seyffert et al., 2016).

Based on previous research on CBT-i and internet-delivered CBT-i (iCBT-i), mentioned above, it has the potential to also be effective in bereaved parents; however, as bereaved parents is a burdened group with a lot of distress, the feasibility and efficacy of iCBT-i needs to be examined in this specific population. This is the first study to examine iCBT-i in a randomized controlled trial in bereaved individuals. This study examined the feasibility and preliminary efficacy of iCBT-i in bereaved parents 1 to 5 years after the loss of a child to cancer. A secondary aim was a preliminary examination of the effect of the insomnia treatment on secondary outcomes including prolonged grief, depression, anxiety, posttraumatic stress, and grief rumination.

## 2 Materials and methods

### 2.1 Study design

A two-armed randomized control trial with iCBT-i versus active control. The study was approved by the regional ethics review board in Stockholm, Sweden (protocol #2015/2183-31/5). This trial was registered at ClinicalTrials.gov, (registration ID #NCT02886052).

### 2.2 Participants and procedure

**Fig. 1:**
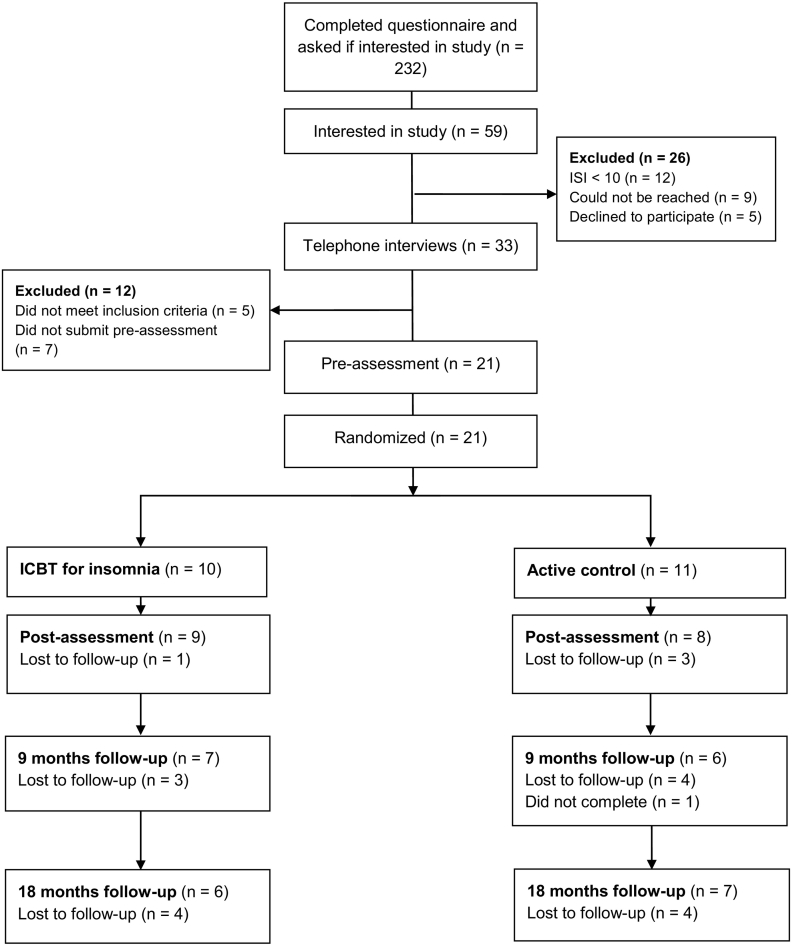
Participant flow through the study.

The present study is part of a larger project on parents of children who were diagnosed with a malignancy before the age of 17 years and died due to the malignancy before the age of 25 years, between 2010 and 2015. Cancer-bereaved parents were invited to take part in a Swedish nationwide postal survey, which has been described in greater detail in Pohlkamp et al. (2018) and Sveen et al. (2019). Of 512 eligible individuals, 372 consented to participate and received the survey, 63 declined to participate and 76 parent could not be contacted. Two hundred and thirty-two parents filled out the survey, which included a question on interest in receiving more information about the present study. A total of 59 individuals indicated such interest. Initial screening was made based on the postal survey, i.e., scoring ≥10 on the Insomnia Severity Index, with 47 participants fulfilling this criterion. They were sent an information letter and contacted by telephone. Twelve parents scored <10 on ISI in the postal survey and were sent a letter explaining that they did not meet the initial inclusion criterion. Of the 47 participants, nine could not be reached and five declined participation (Fig. 1), resulting in 33 participants being interviewed.

#### 2.2.1 Structured telephone interview

The structured telephone interview was based on the clinical interview described by Morin and Espie (2003), translated into Swedish, and with the addition of DSM-5-critera for insomnia (American Psychiatric Association, 2013). The interview also included questions regarding inclusion and exclusion criteria, and was structured with supporting questions and decision algorithms to facilitate use by inexperienced interviewers. Inclusion criteria were: insomnia disorder in accordance with the criteria of the Diagnostic and Statistical Manual of Mental Disorders, Fifth Edition (DSM-5, American Psychiatric Association, 2013), more than 10 points on the Insomnia Severity Index (ISI) (Morin, 1993), and ability to read and write in Swedish. Exclusion criteria were: other comorbid sleep disorders requiring other treatment (sleep apnea or narcolepsy), ongoing alcohol or drug abuse, comorbid disorders directly contraindicative of essential interventions in insomnia treatment (e.g., bipolar disorder), and night-shift work. Initial sleep medication use was unrestricted, but participants were recommended to stabilize their dose during treatment, with the option of tapering. Thirty-three individuals were interviewed via telephone; five did not meet the inclusion criteria, while 28 did and consented to participate in the study. Seven individuals dropped out of the study prior to randomization, and 21 participants filled out the baseline assessment online and were then randomized.

#### 2.2.2 Randomization and assessment points

The participants were randomized to either the intervention group or the active control group by an assistant not otherwise involved in the study, using www.random.org, a true random number source, and randomization of clusters of different sizes. In one case, both parents of a deceased child participated; they were assigned to the same group and counted as one individual in the randomization. Ten parents were randomized to the intervention group and eleven to the control group.

Assessment points were before the treatment, post-treatment and at one month (interview), 9 and 18 months after treatment.

#### 2.2.3 Intervention

The iCBT-i was based on well-established CBT models and techniques, and included active support from therapists during 9 weeks. It consisted of a set of modules on a secure website that only the participants and their therapists could access. Each module included text to read, questions to answer, behavioral assignments, work sheets, and a sleep diary to be filled out throughout treatment. The modules were: introduction and facts about sleep; introduction to CBT for insomnia and sleep hygiene; education on sleep medication and how to quit; sleep restriction and stimulus control; stress management; managing fatigue; handling negative thoughts about sleep; and planning ahead. The participants were supposed to complete about one module a week. Each module ended with a homework assignment consisting of a set of structured, often free-text, questions to be sent to the therapist via the secure messaging system on the website. The therapist would review answers, work sheets and the sleep diary, give written feedback within two weekdays, and give the participant access to the next module. The participants could ask their therapist questions via the messaging system, and these messages were to be answered within two weekdays. Therapist adherence to the treatment protocol was ensured through the internet-delivered format and continuous supervision. There were two therapists, one who was in the final year of the Swedish 5-year university program for clinical psychologists and had training in CBT, and one who had a Bachelor of Science in psychology and had training in CBT. The therapists received initial training in the CBT-i program and weekly supervision.

#### 2.2.4 The active control group

Participants received a short booklet with psychoeducation on sleep, sleep disturbances, setting treatment goals, sleep hygiene, stress management, and mindfulness via the secure website. They could download the booklet if wanted. They had no contact with the therapist. It was not checked if the participants read the booklet or not.

### 2.3 Feasibility – adherence, satisfaction, evaluation of the treatment's strengths and weaknesses, and adverse events

To assess adherence, the numbers of completed modules and homework assignment reports were measured. The numbers of messages between the participants and therapists were also measured.

In the intervention group, participants' satisfaction with treatment was assessed using the Client Satisfaction Questionnaire (CSQ-8) (Larsen et al., 1979) at post-treatment. The CSQ-8 contains 8 items and has a total score ranging from 8 to 32.

#### 2.3.1 Subjective/open-ended evaluation of the treatment's strengths and weaknesses, and adverse events

One month after treatment, the participants from both groups were interviewed via telephone. Adverse events were assessed through the question “Did the treatment have any negative effects?” Participants who answered yes were asked to expand upon this. They were also asked if the treatment had any positive effects and, if so, asked to expand upon this. Further, they were asked about their perceptions of the weaknesses and strengths of the treatment, if there was anything missing or if any part of the treatment was especially useful. Participants in the intervention group who had ended the treatment in advance were asked why they had done so.

### 2.4 Efficacy – symptom measures

#### 2.4.1 Primary symptom measure

The Insomnia Severity Index (ISI) (Morin, 1993) was used to measure self-reported insomnia severity. The psychometric properties of ISI are adequate and it is sensitive to change (Bastien et al., 2001), including when used online (Thorndike et al., 2011). It consists of 7 items, partly corresponding to the symptoms in DSM-5, which assess the severity of sleep onset and sleep maintenance difficulties, sleep pattern satisfaction, interference with daily functioning, noticeability of impairment attributed to the sleep problem, and degree of concern or distress caused by the sleep problem. Treatment response and remission rates were also assessed (Morin et al., 2009): treatment responders were those whose scores decreased by 8 points or more from pre-treatment to post-treatment or follow-up, and remitters were those whose absolute ISI score at post-treatment or follow-up was below 8.

#### 2.4.2 Secondary symptom measures

##### 2.4.2.1 Parent health

The Prolonged Grief Disorder-13 (PG-13) was used to assess symptoms of prolonged grief (Prigerson et al., 2009); it contains 11 items covering cognitive, behavioral, and emotional symptoms, one duration item, and one impairment item. A study of the Swedish PG-13 has shown satisfactory psychometric properties (Pohlkamp et al., 2018). The Montgomery-Åsberg Depression Rating Scale (MADRS) (Montgomery and Asberg, 1979) was used to assess depressive symptoms; it contains 9 items. The PTSD Checklist for DSM-5 (PCL-5) was used to assess symptoms of post-traumatic stress disorder (PTSD) (Weathers et al., 2013); it contains 20 items corresponding to the symptoms in the DSM-5. A study of the Swedish PCL-5 has shown adequate psychometric properties (Sveen et al., 2016). The Generalized Anxiety Disorder-7 (GAD-7) was used to assess self-reported generalized anxiety disorder symptoms (Spitzer et al., 2006); it consists of 7 items. The Utrecht Grief Rumination Scale (UGRS) (Eisma et al., 2012) was used to measure grief-specific rumination; it contains 15 items that measure different aspects of grief rumination. A study of the Swedish UGRS has shown that it has satisfactory psychometric properties (Sveen et al., 2019).

### 2.5 Statistical analyses

Data analysis was performed using the IBM SPSS version 25.0. Demographic, loss-related baseline data, responses, and remission rates were compared using Mann-Whitney *U* tests and chi-squared tests, and statistical significance was set at *p* < 0.05. Fisher's exact test was used when the expected count of one or more cells was below five. All outcome analyses were performed on an intention-to-treat basis, i.e., including measures from all patients, regardless of their adherence to treatment. To examine the program effects on the primary and secondary outcome variables, Generalized Estimating Equations (GEEs) with a linear scale response and autoregressive (first order) correlation matrix were used. Intervention group, measurement timepoint, and the interaction of group and measurement time point were chosen as factors/covariates. GEEs use all available observations instead of using only participants with complete data for all time points, and are thus suitable for intention-to-treat analyses when there are missing data. A sensitivity analysis of the main outcome, ISI, was performed using a GEE with missing data replaced by the last observation carried forward. For effect sizes, Cohen's *d*s with pooled standard deviations were calculated using observed data.

## 3 Results

### 3.1 Participants and missing data

Twenty-one bereaved parents were included in the study: 14 mothers and 7 fathers. Demographic variables of the deceased children and their parents are presented in Table 1 and missing data are presented in the flowchart (Fig. 1).

**Table 1:** Characteristics of the parents and their children.

	Intervention (n = 10)	Control (n = 11)
Parent characteristics		
Female, n (%)	7 (70%)	7 (64%)
Age in years (SD)	49.9 (5.8)	45.6 (5.5)
Level of education, n (%)		
High school	3 (30%)	9 (82%)
University	7 (70%)	2 (18%)
Married or partnered, n (%)	8 (80%)	8 (73%)
Employment status, n (%)		
Employed or student	7 (70%)	10 (91%)
Sick leave	1 (10%)	1 (9%)
Unemployed	2 (20%)	–

Child characteristics		
Female, n (%)	5 (50%)	4 (36%)
Age at death, years (SD)	11.2 (4.7)	12.8 (4.5)
Time since death, years (SD)	3.1 (1.7)	2.8 (1.5)

Note: There were no significant differences between the intervention and control group for any variables displayed in the table, except for education (*X*^2^ = 5.7, *p* < 0.05).

### 3.2 Feasibility – treatment adherence, treatment satisfaction and adverse events

#### 3.2.1 Treatment adherence

One participant dropped out of treatment immediately and did not complete or read any modules, and one participant completed the first module and then dropped out. One individual, after receiving the first module, did not complete any homework assignments or modules.

An average of 4.4 (SD = 2.8) of the 9 modules were made available and the participants completed the homework for 3.5 (SD = 2.9) of the modules on average. The homework for the module on sleep restriction and stimulus control, which is considered the core of the treatment, was completed by 7 participants. On average, 18.6 messages were sent from each participant to the therapist and 15.4 messages were sent from the therapist.

#### 3.2.2 Treatment satisfaction

Seven participants in the intervention group completed the CSQ-8 at post-treatment, with a total mean score of 24.1 (SD = 5.5) from a maximum score of 32, which represents a “good” overall level of satisfaction (Larsen et al., 1979). Most participants reported that the quality of treatment was good and were satisfied with the amount of help received. Overall, four participants were very satisfied with the treatment, while two were indifferent, and one person was quite dissatisfied. Lack of time to complete the modules and treatment being too time-consuming were the most common comments. All responded yes to the question if they would recommend the treatment to a friend.

#### 3.2.3 Subjective/open-ended evaluation of the treatment's strengths and weaknesses, and adverse events

All members in both groups were contacted for a telephone interview; 8 from the treatment group and 5 from the control group answered. In the treatment group, 7 participants reported that the treatment had positive effects. Most participants stated that they were sleeping better. Other positives were more knowledge about sleep, using relaxation techniques, and sleeping without interruptions. One person, who was dissatisfied, stated that they had not experienced any direct positive effects and that they lacked the time needed for completing the treatment. In response to the question “Is there anything especially good about the treatment?”, 4 stated that the support and responses from the therapist were important and 2 thought the knowledge and suggestions from the treatment had been helpful. Reasons for ending the treatment before completing were that sleep restriction was too difficult and that the treatment was too intensive and time-consuming. No one in the intervention group had experienced any negative effects of treatment.

One participant in the control group stated that the sleep booklet had a slight positive effect, while no participant reported any negative effects of the sleep booklet.

### 3.3 Primary symptom outcome – insomnia severity

Descriptive data for insomnia severity at each time point are shown in Table 2. At baseline, there was no statistically significant difference in insomnia severity between the intervention group and the control group (Mann-Whitney *U* = 48, *p* > 0.05). A group comparison of change in insomnia severity over all four timepoints revealed an overall statistically significant interaction effect in favor of the intervention group (Wald χ^2^ = 30.0, *p* < 0.001). The results were the same in the sensitivity analyses (Wald χ^2^ = 25.5, *p* < 0.001). The intervention group showed a steady decrease in insomnia symptoms and had statistically significantly lower levels of such symptoms at post-treatment (*d* = 1.6) and at follow-up assessments (9 months, *d* = 3.6; 18 months, *d* = 4.1) compared with at baseline. The active control group had fluctuating levels of insomnia symptoms across the three timepoints after treatment and symptom levels were statistically significantly lowered only at post-treatment (*d* = 1.1) and the 18-month follow-up (*d* = 1.5) compared with at baseline. When comparing absolute insomnia levels at each timepoint, the intervention group had statistically significantly lower levels at the 9-month follow-up (*d* = −1.5), but not at post-treatment (*d* = 0.1) or the 18-month follow-up assessments (*d* = −0.6).

**Table 2:** Mean scores and standard deviations (SD) observed and effect sizes (Cohen's *d*) of within- and between-group differences for primary and secondary outcome measures.

	Intervention (n = 10)				Control (n = 11)				Between-group
	n	Mean	SD	*d*	n	Mean	SD	*d*	*d*
Insomnia (ISI)									
Pre-treatment	10	20.0	2.9		11	18.5	5.0		
Post-treatment	9	13.2	5.9	1.6⁎	8	12.6	6.1	1.1⁎	0.1
9-Month follow-up	7	8.0	4.3	3.6⁎	7	16.0	7.6	0.3	−1.5⁎⁎
18-Month follow-up	6	8.2	3.4	4.1⁎	7	10.7	5.9	1.5⁎	−0.6

Prolonged grief (PG-13)									
Pre-treatment	10	34.7	7.4		11	40.3	6.8		
Post-treatment	8	32.5	9.6	0.3	8	34.3	5.8	1.0⁎	−0.2
9-Month follow-up	7	29.0	7.5	1.0	6	33.8	9.6	0.9⁎	−0.6
18-Month follow-up	6	23.3	4.9	2.6⁎	7	34.6	7.3	0.9⁎	−1.9⁎⁎

Depression (MADRS)									
Pre-treatment	10	20.7	6.9		11	22.5	8.0		
Post-treatment	8	16.4	7.2	0.7⁎	8	17.6	7.3	0.7	−0.2
9-Month follow-up	7	12.1	5.6	1.4⁎	6	20.3	7.8	0.3	−1.3⁎⁎
18-Month follow-up	6	9.0	5.0	2.0⁎	7	17.3	6.9	0.7⁎	−1.5⁎⁎

Anxiety (GAD-7)									
Pre-treatment	10	8.0	5.8		11	10.7	6.1		
Post-treatment	8	7.4	3.3	0.1	8	6.6	4.1	0.8⁎⁎	0.2
9-Month follow-up	7	3.7	2.7	1.0⁎⁎	6	11.7	4.8	−0.2	−2.3⁎⁎⁎
18-Month follow-up	6	4.0	4.0	0.8⁎⁎	7	9.0	5.5	0.3	−1.1⁎

Posttraumatic stress (PCL-5)									
Pre-treatment	9	31.0	15.2		11	31.0	16.8		
Post-treatment	8	23.5	12.5	0.6⁎	8	23.5	14.9	0.8⁎	−0.4
9-Month follow-up	7	18.4	15.1	0.9⁎	6	32.7	14.0	0.5⁎	−1.1⁎
18-Month follow-up	6	10.8	8.2	1.7⁎	6	24.3	11.9	1.1⁎	−1.5⁎⁎

Grief rumination (UGRS)									
Pre-treatment	10	43.1	13.3		11	54.6	10.9		
Post-treatment	8	41.6	12.0	0.1	8	45.8	14.1	0.8⁎⁎	−0.3
9-Month follow-up	7	37.1	12.0	0.5	6	48.5	15.1	0.6	−0.9
18-Month follow-up	6	26.0	4.9	1.7⁎	7	45.9	10.0	0.9⁎⁎⁎	−2.7⁎⁎

Note: ISI = Insomnia Severity Index; PG-13 = Prolonged Grief Disorder-13; MADRS = Montgomery-Åsberg Depression Rating Scale; GAD-7 = Generalized Anxiety Disorder-7; PCL-5 = PTSD Checklist for DSM-5; UGRS = Utrecht Grief Rumination Scale.

⁎ *p* < 0.05.

⁎⁎ *p* < 0.01.

⁎⁎⁎ *p* < 0.001.

There were no statistically significant differences between the intervention and control groups concerning treatment response (χ^2^ = 0.04–3.90, *p* > 0.05) or remission rates (χ^2^ = 0.3–4.7, *p* > 0.05) at any assessment point. In the intervention group, there were 2 (20%) remitters and 3 (30%) responders at post-treatment, 5 (50%) remitters and 3 (30%) responders at the 9-month follow-up and 2 (20%) remitters and 5 (50%) responders at the 18-month follow-up. In the control group, there were 1 (9%), 1 (9%), and 3 (27%) remitters, and 2 (18%), 1 (9%), and 2 (18%) responders, respectively, at the corresponding timepoints.

### 3.4 Secondary symptom outcomes – symptoms of prolonged grief, depression, anxiety, posttraumatic stress and grief rumination

Descriptive data for the secondary outcomes for each measurement time point are shown in Table 2. There were no differences between the groups at baseline on the secondary measures of prolonged grief, depression, anxiety, and posttraumatic stress, except that grief rumination was more common in the control group (Mann-Whitney *U* = 85.5, *p* > 0.05).

Group comparisons over all four timepoints revealed no overall statistically significant interaction effects for any of the secondary outcome measures (Wald χ^2^ = 3.2–7.3, *p* > 0.05), except for the anxiety measure (χ^2^ = 15.36, *p* < 0.01). However, there was a general trend in the treatment group for having a better outcome than the control group when comparing the absolute levels at different timepoints (see Table 2).

## 4 Discussion

This study showed positive results regarding feasibility, acceptability, and preliminary efficacy of iCBT-i in bereaved parents 1 to 5 years after the loss of a child to cancer.

The study provided evidence for feasibility and acceptability, with the treatment group reported satisfaction with the treatment and stating that they would recommend it to a friend. The majority reported that it had positive effects and no one reported any negative effects. However, two individuals had wanted more support during sleep restriction. Although the treatment was reported by some parents to be time-consuming, the parents reported that they were sleeping better. In addition, several participants appreciated the support from the therapist.

The score for satisfaction with the iCBT-i, as reported in the CSQ-8, was 24.1, which is good and comparable to previous studies on internet-based CBT-i, which reported between 23.1 and 27.3 for the same measure (Blom et al., 2015a; Blom et al., 2015b; Kaldo et al., 2015). The participants in this study had completed a smaller number of modules, 38%, as compared with those in previous studies, where completion ranged from 68% to 82% (Blom et al., 2015a; Blom et al., 2015b; Kaldo et al., 2015), and a smaller number of homework assignments: 39% compared with 68% in Kaldo et al. (2015). However, 70% of the individuals in this study completed the main assignments on sleep restriction and stimulus control. Overall, the comparison suggested a somewhat lower level of adherence in this study. There could be many reasons for this. The bereaved parents in this study had high symptoms levels, such as depression, posttraumatic stress and prolonged grief, compared with a larger overlapping sample by Pohlkamp et al. (2019). This suggests that the parents in this study were heavily burdened by their loss, with a lot of comorbid symptoms, and might have found it difficult to complete this version of CBT-i. The initial motivation might have been lower in this study, possibly influenced by the recruitment method: parents were offered treatment due to their participation in a survey, without having actively sought it, as they would have in regular care or when responding to an advertisement for a clinical trial. Also, the two therapists were rather inexperienced with both internet-delivered treatment and CBT for insomnia. However, they had university-level training in CBT and were supervised by a licensed psychologist. Furthermore, the numbers of messages sent between the therapists and the participants were at a similar level as those reported by Kaldo et al. (2015) and Blom et al. (2015a).

The treatment group improved significantly, with large within-group effect sizes for the primary outcome, insomnia severity. There was a pattern of an overall greater reduction of insomnia severity for the treatment group if seen over all three follow-ups. However, the between-group effect size at post-treatment was very small for the primary outcome and there were rather large fluctuations over the follow-up time-points, most probably due to the small number of participants. The secondary outcomes (prolonged grief, depression, posttraumatic stress, anxiety, and grief rumination) showed positive results with small to large effect sizes, although the overall change in symptoms compared with the control group was not statistically significant, except in the case of anxiety. The active control group also improved significantly, with small to large effect sizes for the primary and secondary outcomes. The treatment group had significantly lower levels of psychological symptoms than the control group at the 18-month follow-up, except for insomnia severity, where the difference was not statistically significant.

The positive effect of iCBT-i in adults is well-known (Seyffert et al., 2016), but this is the first study examining its effect in bereaved parents. The main finding is that the insomnia treatment ameliorated insomnia severity in bereaved parents. Interestingly, it was indicated that the comorbid symptoms improved in the long term as well. The treatment group had significantly lower symptom levels than the control group at the 18-month follow-up, which indicates that the effect was not due merely to symptoms levels abating in a natural bereavement process. The effects included an improvement in grief rumination, which has not been examined previously. Though there is a well-established association between rumination and sleep disturbances (Pillai and Drake, 2015), only one previous study has examined the association between grief rumination and insomnia symptoms, showing a moderate association (Sveen et al., 2019).

Our results are in line with previous studies, suggesting that treating insomnia with CBT-i is possible even when comorbid conditions are present and, furthermore, that it seems to reduce the comorbid symptoms. For example, treating insomnia in adults with comorbid depression improves not only insomnia severity, but also symptoms of depression (Blom et al., 2015a; van der Zweerde et al., 2019), and treating posttraumatic stress disorder with CBT and CBT-i for sleep disturbances reduces symptoms of posttraumatic stress (Ho et al., 2016). However, this has not been examined in bereaved individuals with prolonged grief disorder. A tentative conclusion from this study is that treating insomnia reduces symptoms of prolonged grief disorder.

### 4.1 Study limitations and strengths

A limitation of this study was the small sample size and low statistical power. The study needs to be replicated in larger samples. We had aimed for a sample size of 60 participants, but could not recruit that many with the current recruitment method. Only 59 individuals out of the 232 who responded to the survey (i.e. 25%) signaled an interest in receiving more information about the current study. This indicates that the demand for this type of treatment, i.e. in the internet self-help format and not focused on the primary problem (bereavement), could be rather low in the group we targeted and that future studies should use a broader recruitment or offer the treatment in another context. In addition, future studies could compare iCBT-i with face-to-face CBT-i to examine whether face-to-face treatment is more feasible in this population. Also, lower response rates at the follow-ups make the results less certain. On the primary outcome, the attrition was 10–40% in the intervention group and 27–36% in the control group. Hence, the results should be interpreted with caution, although our sensitivity analyses on the primary outcome did not indicate different results. A limitation is that may be a recall bias of adverse events as the interview was conducted one month post-treatment. A strength is that the risk for so-called therapist drift, i.e., that the therapist changes the contents of the treatment along the way, was minimized, as the therapeutic content in the intervention largely consisted of standardized self-help material. Another strength was that the active control group never received the CBT-i treatment; hence, we could compare the groups at the long-term follow-ups.

### 4.2 Clinical implications

Many bereaved parents have multiple psychological symptoms, including sleep problems, for many years after their loss. The present study showed that iCBT-i appears to be feasible and acceptable for bereaved parents who agree to try this treatment format. However, they may need support and encouragement from a therapist, especially when it comes to sleep restriction, which can be very demanding. Furthermore, this study yielded preliminary results that treating insomnia has a positive effect on insomnia severity and also indicated positive effects on other psychological symptoms, such as prolonged grief, depression, anxiety, posttraumatic stress and grief rumination, in bereaved parents.

Taken together with previous findings (van Straten et al., 2018; Wu et al., 2015), which show that insomnia can be effectively treated despite comorbidity, the results of this study provide support that this is the case also for bereaved individuals with insomnia. Thus, CBT-i could be implemented within bereavement care. Further studies on the effect of CBT-i in bereaved samples are needed both in samples with large comorbid problems and in bereaved individuals with low comorbid symptom levels.

## 5 Conclusions

To our knowledge, this is the first study to examine the feasibility and efficacy of iCBT-i in bereaved parents. The study suggested that the treatment is feasible and there were no reported adverse effects. The results showed preliminary evidence that iCBT-i can effectively alleviate insomnia, and to some extent comorbid problems, in bereaved parents, and could thus be offered to bereaved parents with comorbid symptoms. However, on insomnia severity the effect fluctuated rather much and was very small (*d* = 0.1) at post-treatment, but large at the 9 months follow-up (*d* = 3.6). Further research in larger samples and to clarify the specific relationship between insomnia and the comorbid symptoms is crucial. Given the high levels of insomnia and psychological symptoms in bereaved parents, this burdened group deserves more attention.

## Ethical standards

The authors assert that all procedures contributing to this work complied with the ethical standards of the relevant national and institutional committees and with the Helsinki Declaration of 1975, as revised in 2008.

## Funding details

This work was supported by the 10.13039/501100006313Swedish Childhood Cancer Foundation (grant numbers TJ2015-0021, PR2015-0050) and the regional agreement on medical training and clinical research (ALF) between Region Stockholm and 10.13039/501100004047Karolinska Institutet.

## Declaration of competing interest

The authors declare that they have no known competing financial interests or personal relationships that could have appeared to influence the work reported in this paper.
